# A New Compact Double-Negative Miniaturized Metamaterial for Wideband Operation

**DOI:** 10.3390/ma9100830

**Published:** 2016-10-13

**Authors:** Md. Mehedi Hasan, Mohammad Rashed Iqbal Faruque, Sikder Sunbeam Islam, Mohammad Tariqul Islam

**Affiliations:** 1 Space Science Center (ANGKASA), Universiti Kebangsaan Malaysia, 43600 UKM, Bangi, Selangor, Malaysia; mehedi20.kuet@gmail.com (M.M.H.); sikder_islam@yahoo.co.uk (S.S.I.); 2 Department of Electrical, Electronic and Systems Engineering, Universiti Kebangsan Malaysia, 43600 UKM, Bangi, Selangor, Malaysia; tariqul@ukm.edu.my

**Keywords:** dual band, double-negative metamaterial, effective medium ratio, wide bandwidth

## Abstract

The aim of this paper is to introduce a compact double-negative (DNG) metamaterial that exhibits a negative refractive index (NRI) bandwidth of more than 3.6 GHz considering the frequency from 2 to 14 GHz. In this framework, two arms of the designed unit cell are split in a way that forms a Modified-Z-shape structure of the FR-4 substrate material. The finite integration technique (FIT)-based Computer Simulation Technology (CST) Microwave Studio is applied for computation, and the experimental setup for measuring the performance is performed inside two waveguide ports. Therefore, the measured data complies well with the simulated data of the unit cell at 0-degree and 90-degree rotation angles. The designed unit cell shows a negative refractive index from 3.482 to 7.096 GHz (bandwidth of 3.61 GHz), 7.876 to 10.047 GHz (bandwidth of 2.171 GHz), and 11.594 to 14 GHz (bandwidth of 2.406 GHz) in the microwave spectra. The design also exhibits almost the same wide negative refractive index bandwidth in the major region of the C-band and X-band if it is rotated 90 degrees. However, the novelty of the proposed structure lies in its effective medium ratio of more than 4, wide bandwidth, and compact size.

## 1. Introduction

Metamaterials are artificially electromagnetic materials consisting of periodically arranged metallic components that are smaller than the wavelength of the incident electromagnetic (EM) wave in size. Furthermore, the materials can control electromagnetic wave beams in astonishing ways and exhibit various exterior electromagnetic properties that are not obtainable in the nature. These extraordinary properties unequivocally rely on the geometry of the metamaterial’s atomic structure. Metamaterials with concurrent negative permittivity (*ε* < 0) and permeability (*µ* < 0) are called double-negative (DNG) metamaterials. When the permeability and permittivity of a material are equivalent to zero over a specific frequency range, then it is known as a near-zero refractive index metamaterial (NZRI). However, if the material’s properties exhibit either negative permittivity or negative permeability, it is identified as a single-negative (SNG) metamaterial. Veselago et al. [1] recommended a new type of material that exhibited negative permittivity and negative permeability as well as general characteristics of an electromagnetic wave in 1968. Veselago’s institution stayed quiet for a long time until Pendry et al. [2] presented their summary of a thin-wire configuration that displayed a negative estimation of permittivity (*ε*) in 1996 and a split ring resonator with a negative estimation of permeability (*μ*) in 1999. Later, Smith et al. [3] exhibited a new type of metamaterial that showed simultaneous negative permittivity and permeability, and he completed microwave examinations to test its unusual properties in 2000. Because of these intriguing electromagnetic properties, metamaterials can be utilized as a part of numerous important applications, such as super-lenses, waveguides, filters [4], antenna design [5,6], invisibility cloaking [7], reduction of SAR [8], electromagnetic absorbers, and electromagnetic band gaps [9]. At present, multi-band metamaterials or arrays of metamaterials with wide negative refractive index bandwidths have become a promising methodology for specialists, although very few studies have concentrated on designing such materials [10,11,12]. Intrinsic negative permittivity can be found in some metals, but a natural medium with negative permeability is harder to find. Even using artificial structures, it is relatively difficult to realize negative permeability. As a result, simultaneous negative refractive indexes (*ε* < 0 and *µ* < 0) are very difficult to obtain. Numerous alphabetical metamaterial structures have been proposed for particular applications; for instance, Benosman et al. [13] proposed a double “S-shape” metamaterial in the microwave range, which showed a negative refractive index from 15.67 to 17.43 GHz. Mallik et al. [14] introduced a rectangular “U-shape” metamaterial that displayed left-handed attributes at approximately 5, 6, and 11 GHz for various array structures. Ekmekci et al. [15] presented a “V-shape” metamaterial, and the structure exhibited double-negative attributes at 8.10 GHz. Riwan et al. [16] designed an “F-shape” DNG metamaterial for K- and Ka-band, which had a 2.3-GHz negative refractive index bandwidth from 22.5 GHz to 24.8 GHz. An “S-shape” 15 × 15-mm^2^ chiral metamaterial was designed by Zhou et al. [17] for X- and Ku-band, but the shape’s effective medium ratio was less than 4. Islam et al. [18] proposed an “H-shape” metamaterial for multiband microwave applications, and negative index qualities were seen in the C (bandwidth of 0.5 GHz) and S (bandwidth of 0.3 GHz) bands. The dimensions of the metamaterial unit cell were 30 × 30 mm^2^. Hossain et al. [19] presented two “G-shape” double-negative metamaterials for different unit cells and array sizes, which were appropriate for the S- and C-band. Moreover, Alam et al. [20] designed an 8 × 8-mm^2^ “Hexagonal-shape” double-negative medium metamaterial, for which the refractive index regions were obtained from 1.68 to 3.43 GHz (bandwidth of 1.75 GHz) and 5.04 to 6.0 GHz (bandwidth of 0.96 GHz). 

In this study, the designed Modified-Z-shape structure exhibits resonance at C- and X-bands with a wider negative refractive index bandwidth from 3.482 to 7.096 GHz (bandwidth of 3.61 GHz), 7.876 to 10.047 GHz (bandwidth of 2.171 GHz), and 11.594 to 14 GHz (bandwidth of 2.406 GHz), which is a larger bandwidth than that of [19,20]. Moreover, the proposed structure’s dimension is 10 × 10 mm^2^, which is quite a bit smaller than the metamaterial unit cell presented in [17,18]. As a result, the effective medium ratio of the unit cell is more than 4, and it is suitable for operating the sub-wavelength regime. Moreover, double-negative metamaterial properties appear at 8.79 GHz for unit cells and in case of array configurations at 8.60 GHz. The commercially available electromagnetic simulator CST Microwave Studio has been used to monitor the reflection coefficient (*S*_11_) and transmission coefficient (*S*_21_) to determine the effective permittivity, permeability, and refractive index for the proposed metamaterial structure. The paper is organized in the following manner. Construction of the proposed unit cell is described in Section 2. The methodology is explained in Section 3. An equivalent circuit model is discussed in Section 4. Experimental results are elaborately explained in Section 5, and Section 6 concludes this paper.

## 2. Proposed Unit-Cell Construction

**Figure 1:**
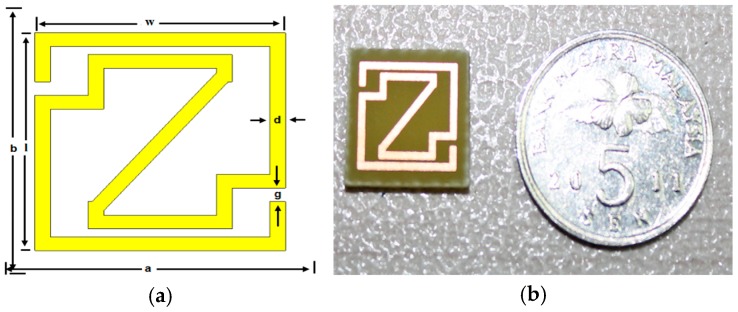
Metamaterial unit cell: (**a**) proposed geometry; (**b**) fabricated geometry.

The diagram of the proposed Modified-Z-shape unit cell configuration is specified in Figure 1. The developed structure consists of a Z-shape split ring resonator of copper with a thickness (*h*) of 0.035 mm. FR-4 is used as the substrate material, which has a dielectric constant of 4.5 and loss tangent of 0.002. The length (*l*) and width (*w*) of the unit cell metal strip are both 8 mm. However, the width (*d*) of every metal strip is 0.5 mm, and the split width (*g*) is 0.5 mm. In this paper, CST Microwave Studio, which is based on the finite integration technique (FIT), is adopted to investigate this design in which an incident electromagnetic wave travels along the positive *z*-axis to the negative *z*-axis. The schematic and a fabricated diagram of the proposed design are illustrated in Figure 1a,b. Table 1 demonstrates, the design parameters of the proposed metamaterial unit cell, where *a*, *b*, *t* are respectively the substrate material width, length and height. 

## 3. Methodology

**Figure 2:**
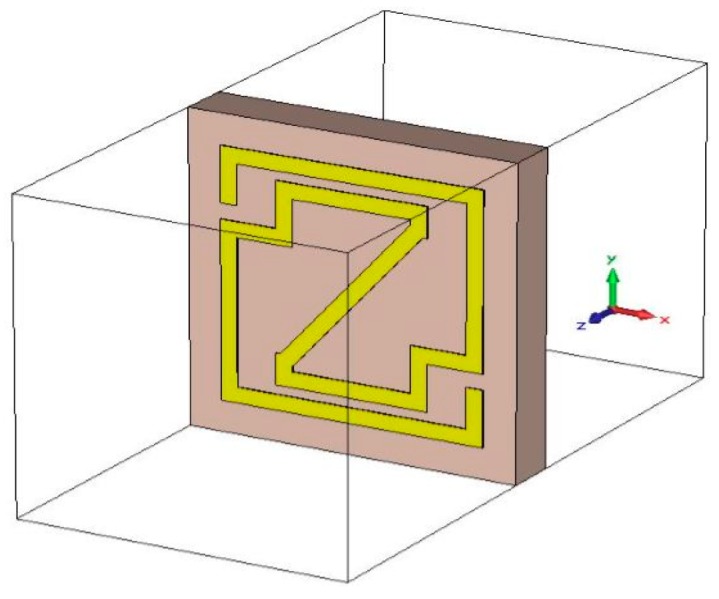
Simulation setup of the proposed Z-shape unit cell for CST Microwave Studio (CST MWS) in a free-space environment.

The commercially available, finite integration technique (FIT)-based CST Microwave Studio was utilized to calculate the scattering parameters. The structure is placed inside the two waveguide ports on the positive and negative *z*-axis. The ideal electric conductor boundary condition was characterized along the dividers perpendicular to the *x*-axis, the dividers perpendicular to the *y*-axis are characterized as perfect magnetic conductor boundaries, and a frequency domain solver was utilized for free-space simulation purposes (shown in Figure 2). Furthermore, a tetrahedral mesh with an adaptive mesh scheme was used for the proposed unit cell and array configuration investigation purposes. The standardized impedance was set to 50 ohms, and the operating frequency was set from 2 to 14 GHz. 

The reflection (*S*_11_) and transmission (*S*_21_) coefficients of the metamaterial unit cell and arrays were investigated to understand the electromagnetic properties of the proposed structures. The restoration of the effective medium ratio depends on the unit cell dimension, and the wave length needs to be less than the working wavelength in the media [21]. Now, the reflection coefficient (Γ) can be determined as follows:

(1) $$\Gamma =\frac{\left(z_{0}-1\right)}{\left(z_{0}+1\right)}$$

$z_{0}$ is the relative impedance for effective permittivity and permeability:

(2) $$z_{0}=\sqrt{\frac{\mu_{r}}{\varepsilon_{r}}}$$

The scattering parameters *S*_11_ and *S*_21_ can be expressed as follows:

(3) $$S_{11}=\frac{\left(1-\Gamma^{2}\right)z}{1-\Gamma^{2}z^{2}}$$

(4) $$S_{21}=\frac{\left(1-z^{2}\right)\Gamma }{1-\Gamma^{2}z^{2}}$$

Now, from *S*_11_ and *S*_21_,

*V*_1_ = *S*_21_ + *S*_11_ (5)

*V*_2_ = *S*_21_ − *S*_11_ (6)

The Nicolson–Ross–Weir approach is utilized to separate the effective permittivity ($\varepsilon_{r}$) and permeability ($\mu_{r}$) from *S*_21_ and *S*_11_. Thus, the effective permittivity and permeability can be calculated by

(7) $$\varepsilon_{r}=\frac{c}{j\pi fd}\times \frac{\left(1-V_{1}\right)}{\left(1+V_{1}\right)}$$

(8) $$\mu_{r}=\frac{c}{j\pi fd}\times \frac{\left(1-V_{2}\right)}{\left(1+V_{2}\right)}$$

However, the Direct Refractive strategy was used for the effective refractive index ($\eta_{r}$), which is calculated from the transmission coefficient (*S*_21_) and reflection coefficient (*S*_11_) [22]:

(9) $$\eta_{r}=\frac{c}{j\pi fd}\times \sqrt{\left\{\frac{{\left(S_{21}-1\right)}^{2}-S_{11}^{2}}{{\left(S_{21}+1\right)}^{2}-S_{11}^{2}}\right\}}$$

**Figure 3:**
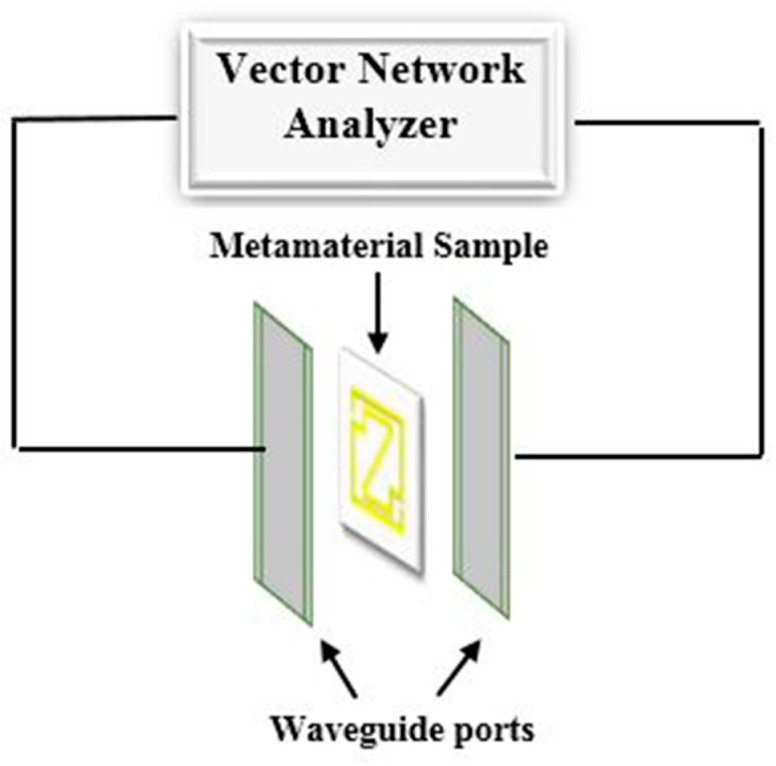
Experimental setup for measuring the S-parameters.

For measurement purposes, a prototype of a 10 × 10-mm^2^ unit cell was fabricated. The measurements were performed in an open-space environment by placing the prototype inside the waveguide shown in Figure 3. An Agilent N5227A vector network analyzer was utilized to determine the scattering parameters of the modified-Z-shape unit cell. Moreover, an Agilent N4694-60001 was used for calibration purposes so that the measurements were performed accurately. 

## 4. Equivalent Circuit Model of the Proposed Unit-Cell

Because the proposed metamaterial structure consists of passive inductive-capacitive (LC) circuits, the resonance frequency (*f*) is

(10) $$f=\frac{1}{2\pi \sqrt{LC}}$$

where *L* and *C* are the aggregate inductance and capacitance of the structure. In the proposed formation, inductances are formed by the metal strip and the capacitances are formed by the splits. Electric resonances are produced by coupling between the gaps and electric fields when the applied electromagnetic wave propagates along the structure. Moreover, magnetic resonances are formed by the coupling between the magnetic fields and loops. Typically, from quasi-static theory, the total capacitance between the gaps is,

(11) $$C=\varepsilon_{0}\varepsilon_{r}\frac{A}{d}\left(F\right)$$

In addition, the total inductance (*L*) for the proposed structure can be estimated from [23,24,25] as

(12) $$L=0.01\times \mu_{0}\left\{\frac{2{\left(d+g+h\right)}^{2}}{{\left(2w+g+h\right)}^{2}}+\frac{\sqrt{{\left(2w+g+h\right)}^{2}+l^{2}}}{\left(d+g+h\right)}\right\}t$$

Therefore, the total capacitance (*C*) can be calculated by

(13) $$C=\varepsilon_{0}\left[\frac{\left(2w+g+h\right)}{2\pi {\left(d+h\right)}^{2}}\ln\left\{\frac{2\left(d+g+h\right)}{\left(a-l\right)}\right\}\right]t$$

where the free-space permeability (*µ*_0_) is 4π × 10^−7^ H/m and the free-space permittivity (*ε*_0_) is 8.854 × 10^−12^ F/m. 

**Figure 4:**
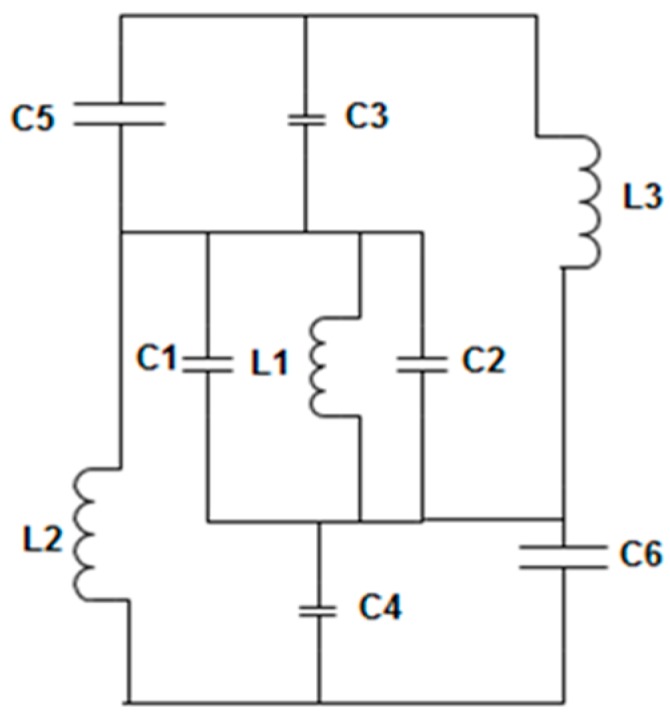
Equivalent LC circuit model of the proposed unit cell.

For wide negative refractive index bandwidth, the series and shunt branch of the unit cell circuit form inductance and capacitance. The splits maintain the capacitive effect and are denoted as C1, C2, C3, C4, and C5 in the circuit. Alternately, the metal strips are responsible for the inductance effect and are named L1, L2, and L3. The addition of more splits in the unit cell structure produces a small phase delay and increases the total capacitance of that metamaterial unit cell. The equivalent circuit of the proposed metamaterial structure is given in Figure 4. 

## 5. Results and Discussion

There are numerous techniques for effective parameter extraction of a metamaterial, such as the Nicolson–Ross–Weir strategy, Direct Refractive Index, etc. In this paper, the electromagnetic properties of the proposed metamaterial are clarified utilizing the real values of the effective permittivity, permeability, and refractive index, which are calculated from *S*_11_ and *S*_21_.

### 5.1. Unit Cell Analysis

**Figure 5:**
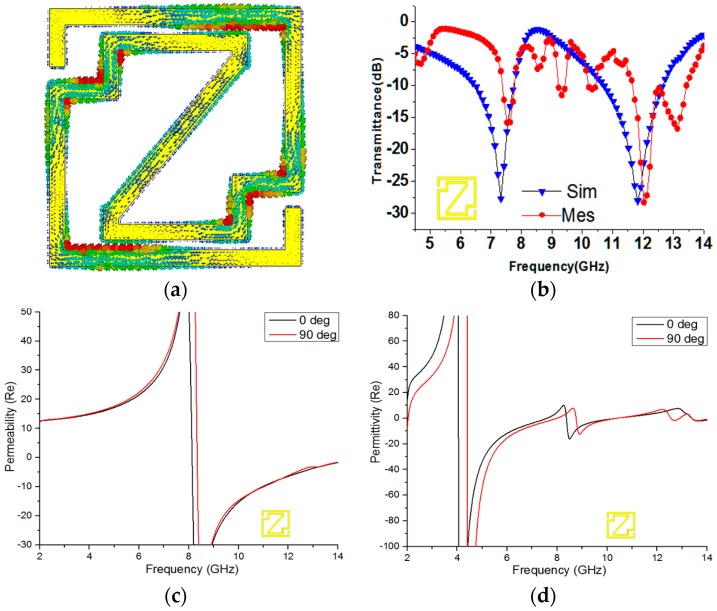
(**a**) Surface current density at 11.84 GHz; (**b**) *S*_21_ curve for the Z-shape unit cell; the real magnitude of (**c**) the effective permeability and (**d**) the effective permittivity of a 0-degree and 90-degree rotated unit cell.

The current density of the unit cell at 11.84 GHz is shown in Figure 5a. The currents flow in opposite directions in the metal strip of the Z-shape unit cell structure because of the dissimilar geometry of the unit cell structure. In the resonator, the opposite current follows the inner and outer surfaces, which causes the stop band at this frequency. In Figure 5b, both the numerical and experimental magnitudes of the S-parameters are shown. The figure shows dual-band resonance at 7.32 GHz and 11.84 GHz in the transmission coefficient (*S*_21_) curve. The measured result presents resonance at 7.53 GHz (C-Band) and 12.02 GHz (X-Band) in the same figure. However, the measured transmittance (*S*_21_) results are slightly shifted and shortened, when compared with the simulated results. This shift usually occurs due to the free-space measurement process or various fabrication errors. 

Figure 5c shows the negative permeability from 8.13 to 14 GHz for 0-degree rotation and from 8.344 to 14 GHz for 90-degree rotation. At a lower frequency, the current can keep pace with the applied field; however, at higher frequencies, the current cannot cope with the applied field and starts to lag in the frequency range when the permeability is negative. Figure 5d shows the negative permittivity from 4.054 to 10.372 GHz and 13.388 to 14.441 GHz for 0-degree rotation and from 4.405 to 7.98 GHz, 8.76 to 10.398 GHz, and 12.556 to 12.803 GHz for 90-degree rotation. Here, it reveals that there is variation between the properties of permeability and permittivity because of the polarization effect due to the interior construction of the materials. When electromagnetic waves enter unequal lattice axes of anisotropic materials, the properties are affected by the polarization inside the material. As a result, the values of permeability and permittivity change due to changes in the design. However, the refractive index curve is also affected by the polarization via the same procedure. 

**Figure 6:**
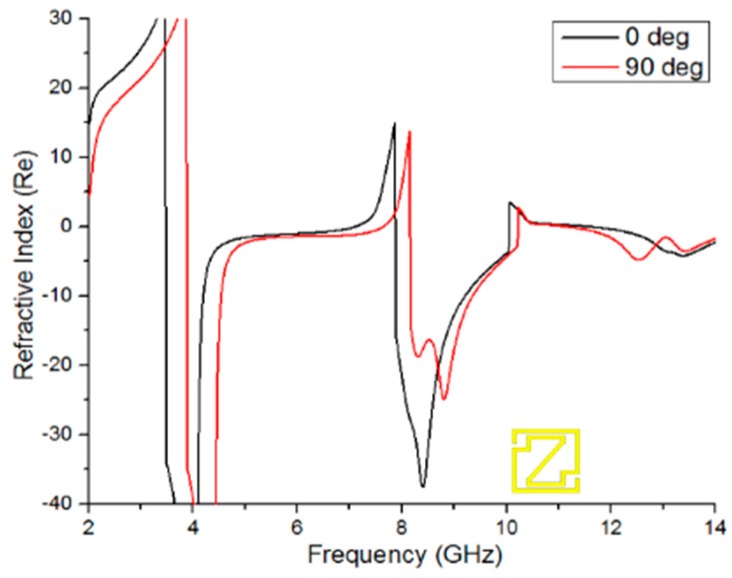
Real magnitude of the refractive index (*η*) vs. the frequency of the 0-degree and 90-degree rotated unit cell.

If the unit cell permittivity and permeability appear negative simultaneously, then the refractive index curve will be negative. Figure 6 shows a negative refractive index from 3.482 to 7.096 GHz (bandwidth of 3.61 GHz), 7.876 to 10.047 GHz (bandwidth of 2.171 GHz), and 11.594 to 14 GHz (bandwidth of 2.406 GHz) for 0-degree rotation and from 3.885 to 7.642 GHz (bandwidth of 3.757 GHz), 8.162 to 10.216 GHz (bandwidth of 2.055 GHz), and 11.152 to 14 GHz (bandwidth of 2.848 GHz) for 90-degree rotation of the unit cell. Therefore, the designed unit cell has characteristics of a double-negative metamaterial because the refractive index, permittivity, and permeability curves have negative peaks at 8.79 GHz, which is shown in Table 2.

### 5.2. Metamaterial Arrays Configurations

Three types of arrays are investigated in this section, and all of the array configurations are called open arrays because the unit cells are not connected with each other. The effective medium parameters of arrays are measured and investigated by 0-degree and 90-degree rotation of the metamaterial structure. 

#### 5.2.1. 1 × 1 Array Analysis

**Figure 7:**
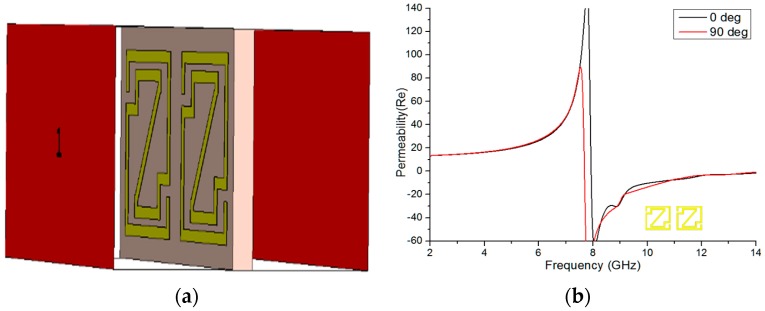

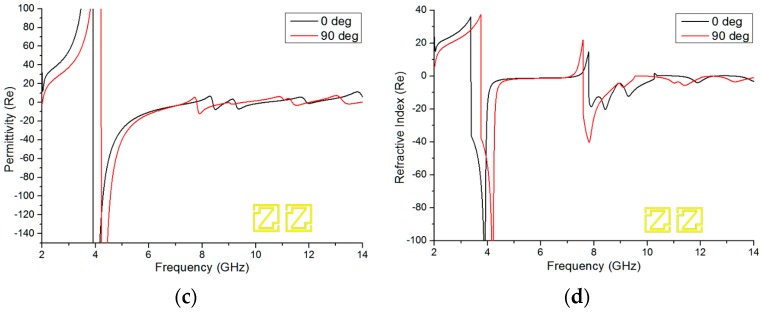
(**a**) 0-degree position of the 1 × 1 array geometry; real magnitude of (**b**) the effective permeability; (**c**) the effective permittivity; and (**d**) the effective refractive index at 0-degree and 90-degree rotations of the 1 × 1 array configuration.

Figure 7a shows the 1 × 1 array geometry at 0-degree rotation. The magnitudes of the effective permeability and permittivity of the Z-shape unit cell are plotted in Figure 7b,c, respectively. In Figure 7b, negative permeability is exhibited from 7.928 to 14 GHz for 0-degree rotation and from 7.688 to 14 GHz for 90-degree rotation. Figure 7c depicts negative permittivity from 3.904 to 7.652 GHz, 8.396 to 8.888 GHz, and 9.036 to 10.34 GHz for 0-degree rotation and from 4.22 to 7.40 GHz, 7.796 to 9.512 GHz, 11.372 to 11.984 GHz, and 13.328 to 13.916 GHz for 90-degree rotation. 

Figure 7d expresses the magnitude of the refractive index (*η*) vs. frequency from 3.38 to 7.34 GHz (bandwidth of 3.96 GHz), 7.80 to 10.256 GHz (bandwidth of 2.456 GHz), 11.132 to 12.524 GHz (bandwidth of 1.392 GHz), and 13.268 to 14 GHz (bandwidth of 0.732 GHz) for 0-degree rotation and from 3.752 to 7.124 GHz (bandwidth of 3.372 GHz), 7.592 to 9.536 GHz (bandwidth of 1.944 GHz), and 9.944 to 14 GHz (bandwidth of 4.056 GHz) for 90-degree rotation. The 0-degree and 90-degree 1 × 1 arrays exhibit almost the same bandwidth from 3 to 10 GHz. In addition, between 7.592 GHz and 10.256 GHz, the negative regions of the refractive index, permittivity and permeability curves display negative peaks. As a result, the proposed array structure appears to be a double-negative metamaterial. 

#### 5.2.2. 2 × 2 Array Analysis

**Figure 8:**
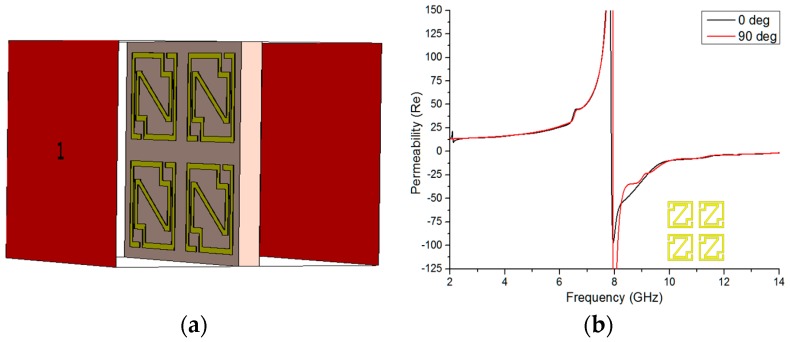

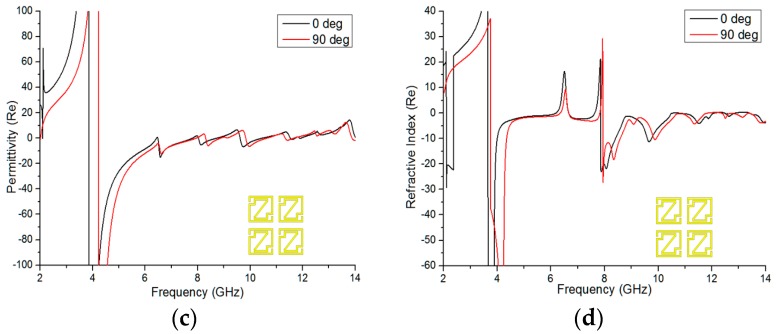
(**a**) 90-degree rotated 2 × 2 array geometry; the real magnitude of (**b**) the permeability; (**c**) the permittivity; and (**d**) the refractive index of the 90-degree rotated 2 × 2 array configuration.

For investigative purposes, the 2 × 2 array configuration was rotated 90 degrees, as seen in Figure 8a. The same methodology was applied to obtain the effective medium parameters from the arrays structure at 0-degree and 90-degree rotations. The real magnitudes of permeability and permittivity of the 2 × 2 array are revealed in Figure 8b,c. Figure 8b’s permeability curve displays a resonance from 7.892 GHz to 14 GHz, which contains a bandwidth of more than 6 GHz, for 0-degree rotation and from 7.94 GHz to 14 GHz, also covering more than 6 GHz, for 90-degree rotation. From Figure 8c, it is evident that the permittivity curves exhibit a negative magnitude from 3.86 to 7.796 GHz, 8.036 to 8.708 GHz, 9.62 to 10.448 GHz, and 11.528 to 11.708 GHz for 0-degree rotation and from 4.22 to 7.928 GHz, 8.312 to 8.864 GHz, 9.836 to 10.628 GHz, and 11.336 to 11.648 GHz for 90-degree rotation. However, two bandwidths, 3.936 GHz for 0-degree rotation and 3.708 GHz for 90-degree rotation, are worth mentioning in the permittivity curve. It was mentioned earlier in this paper that a material’s refractive index curve would be negative if its permittivity and permeability appeared negative simultaneously.

In Figure 8d, the material shows negative peaks from frequencies of 3.656 to 6.176 GHz, 6.668 to 7.628 GHz, 7.856 to 10.544 GHz, 10.772 to 12.08 GHz, and 13.40 to 14 GHz that respectively cover bandwidths of 2.52 GHz, 0.96 GHz, 2.688 GHz, 1.308 GHz, and 0.60 GHz in the microwave regime at 0-degree rotation. The same applies for the frequencies of 3.752 to 6.344 GHz, 6.668 to 7.82 GHz, 7.94 to 11.84 GHz, and 12.356 to 14 GHz that respectively cover 2.592 GHz, 1.152 GHz, 3.90 GHz, and 1.644 GHz bandwidths in the microwave region for 90-degree rotation of the array configuration. Moreover, the 2 × 2 array structure has characteristics of a double-negative metamaterial at 8.60 GHz for both 0-degree and 90-degree rotation. 

#### 5.2.3. 4 × 4 Array Analysis

**Figure 9:**
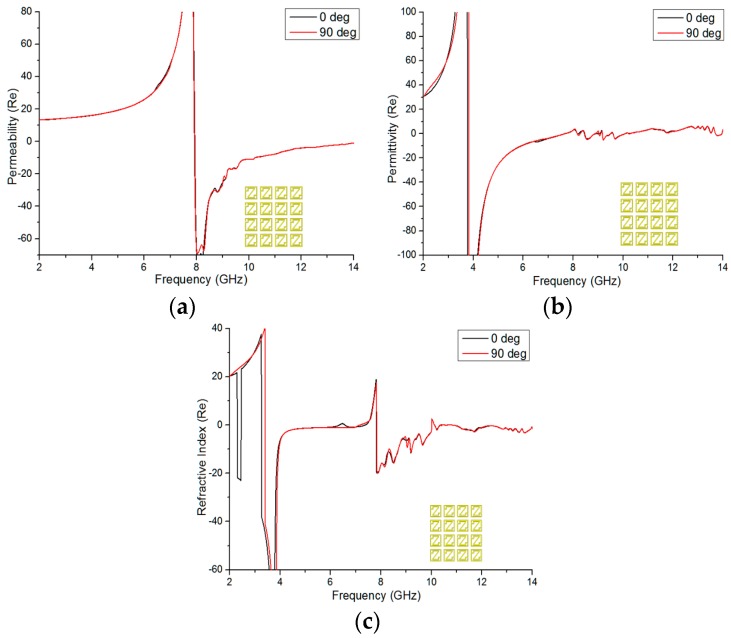
Real magnitude of (**a**) the effective permeability; (**b**) the effective permittivity; and (**c**) the refractive index of the 90-degree rotated 4 × 4 array configuration.

In Figure 9, the permeability, permittivity, and refractive index for 0-degree and 90-degree rotation of the 4 × 4 array structure are depicted. From Figure 9a, negative permeability is shown from 7.94 to 14 GHz, covering almost 6.06 GHz in bandwidth, for 0-degree rotation, and from 7.928 to 14 GHz, covering more than 6 GHz bandwidth, at 90-degree rotation. Moreover, Figure 9b shows negative permittivity from 3.764 to 7.64 GHz, 8.456 to 9.044 GHz, 9.176 to 9.50 GHz, and 9.596 to 10.076 GHz for 0-degree rotation and from 3.824 to 7.664 GHz, 8.468 to 8.876 GHz, 9.044 to 10.268 GHz, and 11.336 to 11.648 GHz for 90-degree rotation. Figure 9c reveals the real magnitude of the refractive index (*η*) vs. frequency from 3.26 to 7.364 GHz, 7.82 to 10.004 GHz, and 10.832 to 14 GHz, respectively covering bandwidths of 4.104 GHz, 2.184 GHz, and 3.168 GHz for 0-degree rotation, and from 3.404 to 7.148 GHz, 7.832 to 10.004 GHz, and 10.832 to 14 GHz, covering refractive index bandwidths of 3.744 GHz, 2.172 GHz, and 3.168 GHz for the 90-degree rotated array structure. Therefore, it is notable here that one bandwidth is greater than 4 GHz and that all of the bandwidths are almost in the same range for both the 0-degree and 90-degree rotations of the array configuration. Finally, the Z-shape structure also exhibits characteristics of a double-negative metamaterial at 8.60 GHz, e.g., in 1 × 1 and 2 × 2 array configurations.

Table 2 represents the value of effective medium parameters for unit cell and array configurations. Here, there are some differences in the parameter values between the 0-degree and 90-degree rotation angles, but the unit cell and arrays configuration are exhibited double-negative characteristics at 8.79 GHz and 8.60 GHz, respectively. Moreover, the effective medium parameter values of the 4 × 4 array structure are almost the same for both the 0-degree and 90-degree rotation angles.

From Table 3, Mallik et al. [14] analyzed the 1 × 1 and 2 × 2 arrays in an orthogonal position. In addition, Islam et al. [18] presented 1 × 1 and 2 × 2 array structures in his framework. Further, Hossain et al. [19] showed a 2 × 2 array configuration at an open and interconnecting position. However, in this paper, 1 × 1, 2 × 2 and 4 × 4 open array configurations were analyzed for 0-degree and 90-degree rotation, which is a new type of analysis from the previous work.

## 6. Conclusions

In this paper, a new Modified-Z-shape negative refractive index (NRI) metamaterial unit cell and array structure are presented, exhibiting a wider bandwidth in the major portion of C- and X-bands with double-negative (DNG) characteristics. For calculation of the transmission (*S*_21_) and reflection (*S*_11_) coefficients of the unit cell and array configurations, CST Microwave Studio was used. The proposed metamaterial is able to exhibit a negative region with a bandwidth greater than 3.6 GHz, which is better than the reference prototype. Similarly, the 4 × 4 array also displays a negative index region with a bandwidth greater than 4 GHz if the proposed structure is rotated 90 degrees. Moreover, C- and X-bands are widely used for satellite communications, especially in many GEO satellites, which are designed to operate in the C-band area. Moreover, the C-band is applicable to modern radio communication, satellite TV networks, and so on. In addition, the X-band is relevant in the military, government, and civil institutions for weather radar monitoring, air and maritime vessel traffic control, defense tracking, and vehicle speed detection for law enforcement. Finally, the proposed double-negative metamaterial unit cell has potential for wider bandwidths in the case of negative refractive index applications in addition to the other metamaterials in the microwave frequency range.

## Figures and Tables

**Table 1:** Design specification of the Z-shape unit cell.

Parameters	Dimensions (mm)	Parameters	Dimensions (mm)
*a*	10	*d*	0.5
*b*	10	*g*	0.5
*l*	8	*t*	1.6
*w*	8	*h*	0.035

**Table 2:** Real values of *ε*, *μ*, and *η* in the resonance frequency band for the unit cell and array configuration at 0-degree and 90-degree rotation.

Structure	Frequency (GHz)	Rotation Angle	Value of Permittivity (*ε*)	Value of Permeability (*μ*)	Value of Refractive Index (*η*)	Refractive Index Bandwidth	Metamaterial Type
Unit cell	8.79	0-degree	−8.35	−34.04	−16.92	3.61 GHz	DNG
		90-degree	−6.43	−34.16	−24.91	3.57 GHz	
1 × 1 Array	8.60	0-degree	−5.45	−29.83	−13.19	3.96 GHz	DNG
		90-degree	−2.31	−35.08	−9.06	4.05 GHz	
2 × 2 Array	8.60	0-degree	−0.68	−45.04	−5.55	2.68 GHz	DNG
		90-degree	−2.63	−34.47	−9.53	3.90 GHz	
4 × 4 Array	8.60	0-degree	−4.17	−31.75	−12.80	4.10 GHz	DNG
		90-degree	−4.66	−31.01	−12.94	3.74 GHz	

**Table 3:** Comparison of the proposed metamaterial array structures with the previous array analysis.

Author Name	Year	MMs Shape	Array Analysis
Mallik et al. [14]	2013	U	1 × 1, 2 × 2 orthogonal array
Islam et al. [18]	2014	H	1 × 1, 2 × 2 array
Hossain et al. [19]	2015	G	2 × 2 open & interconnect array
Proposed Design	2016	Z	1 × 1, 2 × 2, 4 × 4 open array for 0-degree & 90-degree rotation
